# Case of Tetanus Cured by the Oleum Terebinthinæ

**Published:** 1823-02

**Authors:** B. Hutchinson


### Art. V.—*Case of Tetanus cured by the Oleum Terebinthinæ.* By B. Hutchinson, Esq.

John Beedham, a prisoner in the Nottinghamshire House of Correction at Southwell, about thirty years of age, and constitutionally of a delicate and irritable fibre, has been for the last twelve years subject to attacks of epilepsy,—the exciting cause of which he attributed to long exposure to the inhalation of oxygenated muriatic acid gas, in his employment at some extensive bleach-works. On his commitment to the House of Correction, I immediately perceived that the energies of the brain, and his intellectual powers, had, from some cause, been materially enfeebled, and many very severe paroxysms of epilepsy soon pointed out to me the cause. Convulsions frequently attacked his whole frame; a frothy moisture issued from his mouth: he would then remain in a state of the most perfect insensibility, and apparently in a profound sleep. After some duration of this torpor, he would gradually be restored to the power of voluntary motion and to his senses; his memory retaining no traces whatever of his immediately preceding state of epileptic paroxysm. Under the impression that congestion of blood in the vessels of the brain might exert its baneful influence in the production of these symptoms, I began my remedial treatment by local and general bleeding, and by purging with calomel and jalap. I then commenced my curative plan by exhibiting antispasmodics and some preparations of the metallic tonics; under which treatment the paroxysms of my suffering patient were less frequent and much less severe.

On making my daily visit to the prison in the beginning of December, I was informed by one of the turnkeys that Beedham was unable to open his jaws, and that they had been immovably closed since my visit on the preceding day. On entering Beedham's ward, I found the turnkey's account correct, with the addition of a sense of stiffness and pain in the back part of the neck, a considerable spasmodic rigidity of the whole of the muscles of the neck and back, accompanied with pain and uneasiness at the lower part of the tongue, and with some interruption to the facility of swallowing his saliva. He was affected with considerable pain at the lower extremity of the sternum, extending into the back, materially deranging the functions of respiration, from the spasmodic contractions of the diaphragm, and of the muscles subservient to that important office; symptoms strongly threatening that peculiar aspect of the disease termed Prosthotonos. His pulse was 120; his countenance denoting the greatest distress and anxiety; and my prognosis most inauspicious.

I immediately took from his arm about thirty ounces of blood; and, one of the molares of the lower jaw being fortunately wanting, I introduced into his mouth three pills, containing fifteen grains of calomel and two grains of opium. A brisk purging enema, containing one ounce of the oil of turpentine, was administered; and a large blister was applied between his shoulders. On visiting my patient after the lapse of eight hours, the symptoms, instead of showing the least mitigation, were aggravated by an evident increase of muscular rigidity, spasm, and pain. The flexors of the head and trunk became so strongly affected as to balance the extensors, and to keep the head and trunk straight and rigidly extended, incapable of being moved in any way. The enema had produced no effect on the bowels. I immediately resolved upon giving the oleum terebinthinæ a fair trial in this very distressing situation, and directed half an ounce to be given every two hours in gruel.

On the following morning I paid an early visit to my patient, who received me with a cheerful countenance, opening and extending his mouth to show me that he had completely regained the proper command over these muscles. On inquiry, I found that he had taken two ounces of the turpentine; and that, after the second half-ounce had been taken, the spasms began to relax; his pains, consequently, began to abate, and his bowels to be very freely evacuated; and since that period there has not been the least disposition to any return of tetanus. He has suffered several paroxysms of epilepsy, of a much milder character.

*Southwell; December* 1822.

